# GFP–Margatoxin, a Genetically Encoded Fluorescent Ligand to Probe Affinity of Kv1.3 Channel Blockers

**DOI:** 10.3390/ijms23031724

**Published:** 2022-02-02

**Authors:** Kristina R. Denisova, Nikita A. Orlov, Sergey A. Yakimov, Elena A. Kryukova, Dmitry A. Dolgikh, Mikhail P. Kirpichnikov, Alexey V. Feofanov, Oksana V. Nekrasova

**Affiliations:** 1 Faculty of Biology, Lomonosov Moscow State University, 119234 Moscow, Russia; tina.denisova2000@gmail.com (K.R.D.); n.orlov858@yandex.ru (N.A.O.); dolgikh@nmr.ru (D.A.D.); kirpichnikov@inbox.ru (M.P.K.); avfeofanov@yandex.ru (A.V.F.); 2 Shemyakin-Ovchinnikov Institute of Bioorganic Chemistry, Russian Academy of Sciences, 117997 Moscow, Russia; sa-yakimov@yandex.ru (S.A.Y.); kelen.kryukova@yandex.ru (E.A.K.); 3 Emanuel Institute of Biochemical Physics, Russian Academy of Sciences, ul. Kosygina, d. 4, 119334 Moscow, Russia

**Keywords:** Kv1.3 channel, margatoxin, GFP, pore blocker, fluorescent ligand, affinity, neurological disorders

## Abstract

Peptide pore blockers and their fluorescent derivatives are useful molecular probes to study the structure and functions of the voltage-gated potassium Kv1.3 channel, which is considered as a pharmacological target in the treatment of autoimmune and neurological disorders. We present Kv1.3 fluorescent ligand, GFP–MgTx, constructed on the basis of green fluorescent protein (GFP) and margatoxin (MgTx), the peptide, which is widely used in physiological studies of Kv1.3. Expression of the fluorescent ligand in *E. coli* cells resulted in correctly folded and functionally active GFP–MgTx with a yield of 30 mg per 1 L of culture. Complex of GFP–MgTx with the Kv1.3 binding site is reported to have the dissociation constant of 11 ± 2 nM. GFP–MgTx as a component of an analytical system based on the hybrid KcsA–Kv1.3 channel is shown to be applicable to recognize Kv1.3 pore blockers of peptide origin and to evaluate their affinities to Kv1.3. GFP–MgTx can be used in screening and pre-selection of Kv1.3 channel blockers as potential drug candidates.

## 1. Introduction

Voltage-gated potassium channel Kv1.3 is involved in a variety of physiological processes ranging from regulating action potential threshold to migration, proliferation and hormone secretion. The increased attention to the Kv1.3 channel is evoked by its therapeutic potential as a drug target in autoimmune diseases, such as psoriasis, rheumatoidal arthritis, type I diabetes mellitus, and multiple sclerosis [1,2]. Moreover, Kv1.3 is recognized to regulate activation of microglia, and a large line of research focuses on the role of Kv1.3 channel in neuroinflammation inherent in ischemic stroke, Alzheimer’s disease, Parkinson’s disease, and traumatic brain injury [3]. 

Peptide toxins from scorpion venoms, which are distinguished by their high affinity and selectivity for the target potassium channels, are commonly used to study Kv1.3 channel activity. Functionally, these peptides are Kv1.3 channel blockers, which interact with the external vestibule of the channel occluding the ion-conducting pathway of the pore domain [2]. Inhibiting Kv1.3 channels by peptide blockers results in pronounced physiological effects, providing an opportunity to design peptide-based drugs with high selectivity and low off-target effects [4]. Thus, a demand for peptide blockers with biased selectivity towards the target channel guides the ongoing studies of peptide toxins from the natural sources, as well as further characterization of their binding activities.

The main techniques used to study the pharmacological potential of Kv channel blockers are electrophysiological techniques, which allow one to obtain high-content data on the functional activity of ion channels. These techniques are now adapted for high-throughput screening in drug discovery [5]. The high temporal resolution of electrophysiological measurements allows one to study the influence of conformational changes in a channel on the affinity of its interaction with ligands [6,7,8]. 

Other screening formats are being developed, combining the ion flux assays and fluorescent techniques based on membrane-potential-sensitive fluorescent dyes [9,10,11,12,13]. Although these methods are based on indirect measurement of ion-channel activity, they do not require complex equipment and are easier to implement. Noticeably, one major limitation of fluorescence approaches is their low temporal resolution [14].

Fluorescence-based methods are also applicable for use in binding assays, providing a new format to replace a traditional radioligand approach. The classical example is hongotoxin 1 (HgTx1) mutated to HgTx1-A19C and labeled with sulfhydryl-reactive Cy3-, Cy5-, and Alexa-dyes [15]. The fluorescent derivatives of HgTx1 were effective in binding Kv1 channels on rat brain membranes and offered an advantage to visualize Kv1 channels in rat brain sections using fluorescence microscopy. Other examples include Stichodactyla toxin from sea anemone *Stichodactyla helianthus* modified with fluorescein-6-carboxyl, which was used as a molecular probe to detect Kv1.3 overexpressing T lymphocytes by flow cytometry [16], and tetramethylrhodamine-labeled agitoxin 2 (AgTx2), used as a fluorescent probe to search for and study peptide ligands of Kv1 channels [17]. 

The low yield of chemical synthesis, which is the main disadvantage of the abovementioned fluorescently labeled peptides, can be overcome by engineering genetically encoded chimeras comprising a peptide toxin tagged with a fluorescent protein (FP-Tx). The chimeric proteins GFP-OSK1 and RFP-AgTx2, which were designed on the basis of green (GFP) and red (RFP) fluorescent proteins and peptides OSK1 and AgTx2, were demonstrated to retain the activity profile of the parent ligands [18]; however, in the case of GFP-AgTx2, N-terminal position of GFP was found to enhance selectivity of AgTx2 to the binding site of Kv1.3 channel [19]. FP-Tx were shown to be applicable both for the ligand binding studies and the channel imaging purposes. 

A number of FP-Tx is still limited, and properties of newly designed ligands, which are derived from peptide toxins with different affinity and binding specificity require detailed study to fully unlock the potential of these recombinant fluorescent probes of Kv1 channels. 

In the present work we used margatoxin (MgTx) from venom of the scorpion *Centruroides margaritatus* to obtain a GFP-tagged derivative and to study the interaction of GFP–MgTx with the binding site of the Kv1.3 channel. MgTx is an inhibitor of human Kv1.3 and Kv1.2 channels acting at picomolar concentrations [20]. Pharmacological blockage with MgTx was used as one of the tools to reveal the involvement of the Kv1.3 channel in a wide range of physiological processes such as repolarization of the presynaptic action potential and release of neurotransmitters [21,22], neuronal excitability and metabolic signalling pathways in olfactory bulb and anterior piriform cortex [23], and sympathetic control of cardiovascular function [24]. The ability of MgTx to inhibit delayed-rectifier K^+^ current in Jurkat T-lymphocytes [25] as well as to suppress the pro-inflammatory response of T lymphocytes in vivo elicited the role of Kv1.3 in autoimmunity [26,27]. Using MgTx, the modulatory role of Kv1.3 was established in proliferation of neural progenitor cells [28], damage-induced secretion of pro-inflammatory cytokines and chemokines in brain tissue [29], and in the production of reactive oxygen species by microglial cells related to the pathogenesis of Alzheimer’s disease [30].

To characterize a GFP–MgTx interaction with the Kv1.3 binding site, a bioengineering analytical system was applied, which utilizes hybrid channels KcsA–Kv1.3 expressed in the plasma membrane of *E. coli* cells. KcsA–Kv1.3 hybrid is a bacterial KcsA channel bearing a ligand binding site of the human Kv1.3 channel and retaining its pharmacological profile [31,32,33]. We demonstrate that GFP—MgTx has a high affinity to the ligand binding site of Kv1.3 and can be utilized to recognize Kv1.3 blockers using the competitive binding assay as well as to estimate their affinities.

## 2. Results and Discussion 

### 2.1. Design and Properties of GFP–MgTx

**Figure 1:**
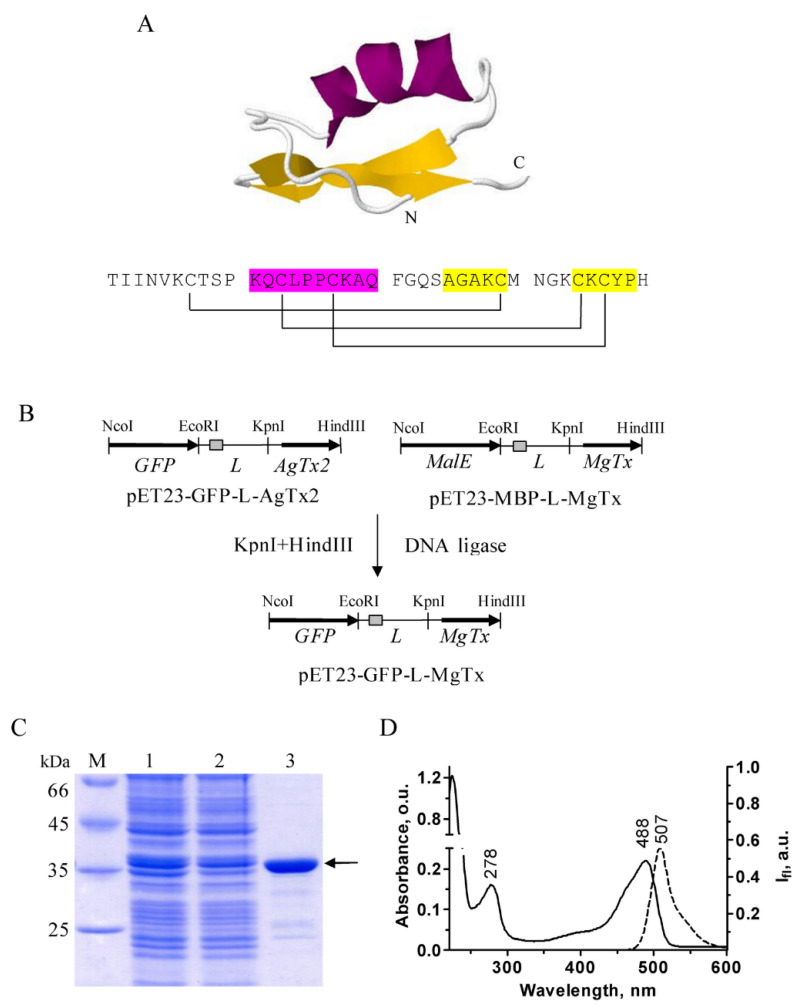
Production and purification of GFP–MgTx. (**A**) 3D structure (PDB ID: 1MTX) and amino acid sequence of MgTx. α-Helix is shown in magenta, β-sheet—in yellow. The pattern of disulfide bond formation is shown with brackets. (**B**) Construction of expression cassette in the plasmid pET23-GFP-L-MgTx. His6 tag is shown as a grey rectangle. *MalE*—a gene encoding maltose binding protein (MBP). L is a linker, which connects a protein tag (MBP or GFP) with the toxin moiety. (**C**) Coomassie-stained 12.0% SDS-PAGE gel that shows the level of total GFP–MgTx biosynthesis in *E. coli* Rosetta-gami B(DE3)pLys (lane 1), cellular lysate after sonication and after subsequent centrifugation (lane 2), and a purified GFP–MgTx after Ni-affinity chromatography (lane 3). M is a protein mass marker. Position of the target protein is shown by an arrow. (**D**) Absorption (solid line) and fluorescence (dashed line) spectra of GFP–MgTx in phosphate-buffered saline. O.u.—optical units.

MgTx is a 39 aa long peptide from α-KTx 2 family of scorpion toxins constituted by a CSa/b (cysteine-stabilized α-helix-β-sheet) structural fold with 6 cysteines that form three disulfide bonds (Figure 1A). According to the 3D structure of MgTx determined by NMR spectroscopy [34], the toxin contains an α-helix and a two-strand antiparallel β-sheet connected by a short loop. The structure is somehow different from that of homologous toxins of the same α-KTx 2 family–noxiustoxin (PDB ID 1SXM) [35] and HgTx1 (PDB ID 1HLY) [15], in which the α/β scaffold consists of a three-stranded β-sheet (with an additional short β-strand at their N-termini).

GFP–MgTx was produced in an *E. coli* expression system in the form of chimeric protein, where a GFP tag was placed N-terminally to the peptide toxin, and GFP was separated from the MgTx sequence by a 51 aa long flexible linker L containing His6 tag (Figure 1B).

The plasmid pET23-GFP-L-MgTx was used to transform *E. coli* strain Rosetta-gami B(DE3)pLysS, which facilitates disulfide bond formation and folding of the target toxin moiety [36]. Induction with a low (0.1 M) concentration of isopropyl β-D-1-thiogalactopyranoside and cultivation of cells at 20 °C resulted in a high (about 90%) solubility of expressed GFP–MgTx. The GFP–MgTx chimera was purified by Ni-affinity chromatography using three wash steps with increasing concentrations of imidazole to improve the purity of the protein preparation to more than 95% (Figure 1C). 

Purified GFP–MgTx was characterized by measuring absorption and fluorescence spectra, which were found to be typical for eGFP (https://www.fpbase.org/protein/egfp/, accessed on 2 January 2022) with absorption maximum at 488 nm and emission maximum at 507 nm (Figure 1D). 

The yield of GFP–MgTx was 30 mg per 1 L of cell culture that provides a significant amount of a fluorescent probe at a relatively low cost. At the same time, the achieved yield of the product is inferior to the yields of GFP–AgTx2 [19] and GFP–HgTx1 [37] (*ca*. 80 mg per 1 L) that may be due to more stringent conditions of Ni-chromatography for GFP–MgTx. 

### 2.2. GFP–MgTx Interaction with the Kv1.3 Binding Site 

Binding of GFP–MgTx to the ligand-binding site of the human Kv1.3 channel was verified using KcsA–Kv1.3 hybrids. For this, KcsA–Kv1.3 were expressed in the plasma membrane of *E. coli* BL21(DE3), and spheroplasts were prepared by removing the cell wall from the freshly grown cells [32,33]. In spheroplasts, the KcsA–Kv1.3 channels become available for interaction with peptide blockers, and for fluorescent ligands, this interaction can be detected with a laser scanning confocal microscopy [32,33]. 

**Figure 2:**
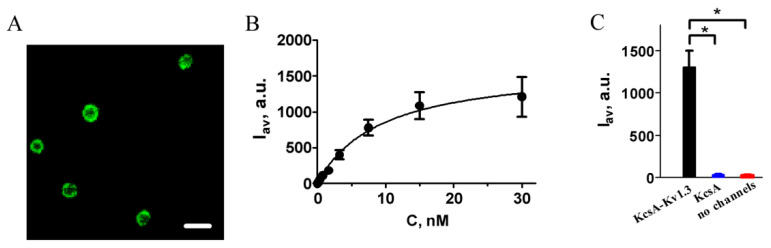
Interaction of GFP–MgTx with the Kv1.3 binding site. (**A**) A typical laser scanning confocal microscopy image of GFP–MgTx (30 nM) bound to spheroplasts expressing KcsA–Kv1.3. Scale bar is 2 µm. (**B**) A typical concentration dependence of the GFP–MgTx binding to KcsA–Kv1.3 at the membrane of *E. coli* spheroplasts. *I_av_* is an average intensity of fluorescence of GFP–MgTx bound to KcsA–Kv1.3 at the surface of a spheroplast (see Materials and Methods for details). Mean *±* SEM is shown, sampling size was >120 cells per point. (**C**) An average intensity of fluorescence of GFP–MgTx (30 nM) associated with the surface of spheroplasts either without channels or expressing KcsA or KcsA–Kv1.3 channels (*—*p* < 0.05). Mean *±* SEM is shown, sampling size was >350 cells per point. The results were averaged over three independent experiments.

**Figure 3:**
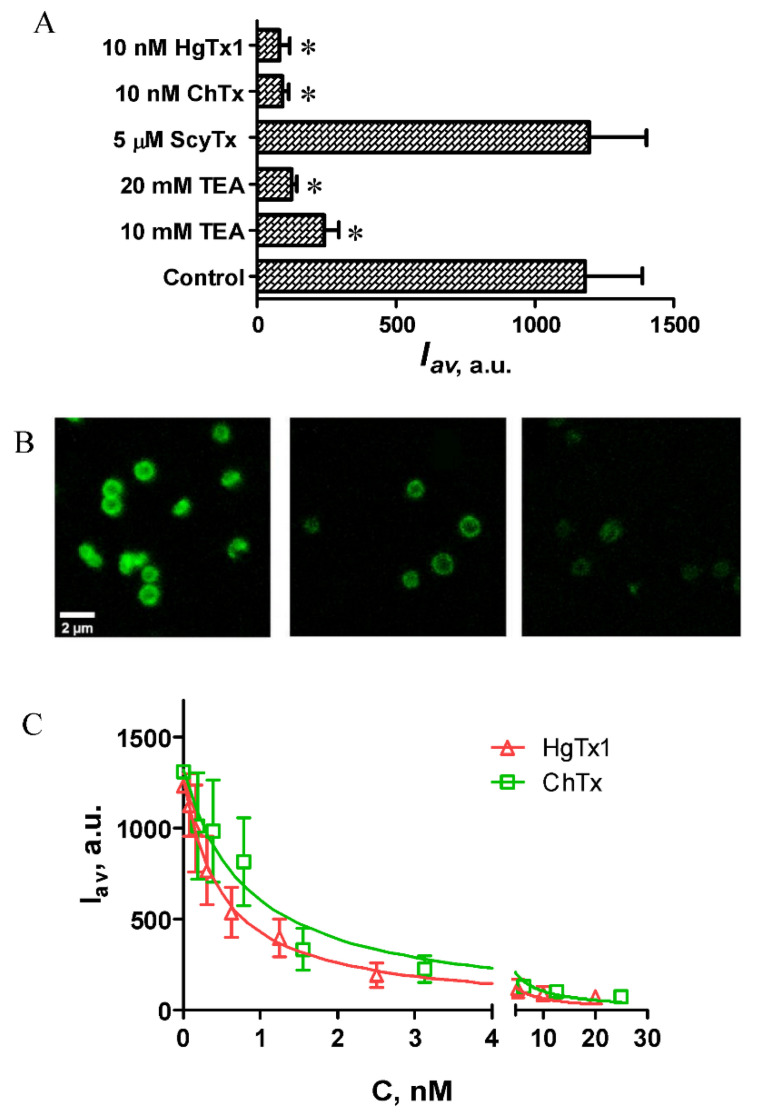
Competitive binding of GFP–MgTx and non-labeled peptide blockers to the Kv1.3 binding site. (**A**) Displacement of GFP–MgTx (30 nM) from the complexes with KcsA–Kv1.3 by HgTx1, ChTx, ScyTx and TEA. *I_av_* is an average intensity of fluorescence of GFP–MgTx associated with the surface of spheroplasts (mean *±* SEM is shown; sampling size was >380 cells per point. The results were averaged over 3 independent experiments; *—*p* < 0.05). (**B**) Typical LSCM images of GFP–MgTx (30 nM) bound to KcsA–Kv1.3 at the spheroplast membrane in the presence of an increasing concentration of a non-labeled peptide blocker, for example, HgTx1. Concentrations of HgTx1 (left to right) are 0, 0.16 and 2.5 nM. (**C**) Typical concentration dependences of the displacement of GFP–MgTx (30 nM) from the complexes with KcsA–Kv1.3 by HgTx1 and ChTx. Mean *±* SEM is shown, sampling size was >120 cells per point.

As imaged with laser scanning confocal microscopy, GFP–MgTx binds to membrane-embedded KcsA–Kv1.3 in the nanomolar concentration range (Figure 2A). This binding is concentration-dependent and saturable (Figure 2B). The binding is specific, as well-known Kv1.3 channel blockers completely inhibited the staining of spheroplasts with GFP–MgTx (Figure 3A), and no binding of GFP–MgTx was detected at the membrane of spheroplasts prepared from non-expressing *E. coli* BL21(DE3) cells, or cells expressing KcsA channel (Figure 2C). As shown earlier, GFP itself is unable to bind to KcsA–Kv1.3 or KcsA, as well as to spheroplasts without heterologously expressed channels [14].

Interaction of GFP–MgTx with the Kv1.3 binding site was characterized quantitatively by analyzing the concentration dependence of GFP–MgTx binding to KcsA–Kv1.3 in the approximation of a single binding site using Equation (1). The dissociation constant *K_d_* of the complex was calculated to be 11 ± 2 nM (mean *±* SEM, averaged over three independent experiments). 

For comparison, radioligand ^125^I-MgTx was shown to bind eukaryotic Kv1.3 channel expressed in CHO cells with *K_d_* value of 0.2 pM in the low-salt buffer, and with *K_d_* of 50 pM in the presence of 100 mM NaCl [38]. Affinity of unlabeled MgTx was ca. 11 pM according to the data of both the radioligand assay [38] and the patch-clamp method [20]. 

Indeed, GFP with the L-linker is a bulk substituent that reduces noticeably the affinity of GFP–MgTx to the Kv1.3 binding site. This seems to be a general property of GFP-tagged peptide blockers. Thus, dissociation constants of the complexes between KcsA–Kv1.3 and previously studied GFP-HgTx1, GFP-L2-AgTx2, His6-GFP-L2-AgTx2 and GFP-OSK1 were 1.1 ± 0.2, 16 ± 4, 10 ± 4, and 1.9 ± 0.4 nM, respectively [18,19,37]. Peptide blockers AgTx2, HgTx1, MgTx, and OSK1 derived from the scorpion venoms have picomolar affinities to Kv1.3 channel (https://kaliumdb.org/, accessed on 2 January 2022). MgTx and HgTx1 belong to the α-KTx 2 family of scorpion toxins, while AgTx2 and OSK1 are members of the α-KTx 3 family. Fusion with GFP reduces the affinity of both α-KTx 2 and α-KTx 3 pore blockers, but the degree of reduction depends on the individual properties of a particular peptide. Nevertheless, the *K_d_* values of the studied GFP-tagged peptide blockers, including GFP–MgTx, which lie in the low nanomolar range, make them attractive fluorescent probes of Kv1.3 channels. 

### 2.3. GFP–MgTx as a Fluorescent Probe to Recognize Kv1.3 Blockers

To estimate applicability of GFP–MgTx for recognition of Kv1.3 blockers, we studied displacement of GFP–MgTx from the complexes with KcsA–Kv1.3 by known pore blockers of Kv1.3 channel, namely, two peptide toxins, ChTx and HgTx1, and a low-molecular-weight blocker tetraethylammonium (TEA), which has millimolar affinity to Kv1.3 [32] (Figure 3). Unlike the majority of small organic Kv1.3-specific blockers, which bind to the internal site of the channel, TEA can bind to both internal and external binding sites [39,40]. The KcsA–Kv1.3-based fluorescent system does not allow one to study compounds that bind Kv-channels from the inner side; however, it enables one to determine whether organic compounds have an affinity for the external binding site and differentially evaluate this affinity. Being a small organic cation, TEA occludes the selectivity filter by the ammonium group. Consequently, affinity of TEA for Kv channel is significantly lower than that of peptide blockers, which are characterized by multipoint interaction with the external binding site. Scyllatoxin (ScyTx), a peptide blocker of KCa2 channels [41] that does not interact with Kv1.3 channel, was used as a control peptide toxin.

GFP–MgTx responded to the presence of Kv1.3 blockers by dissociation from the surface of spheroplasts (Figure 3A,B). Additionally, this response was equally clear in the case of both peptide blockers (ChTx, HgTx1) and TEA. As expected, the binding of GFP–MgTx was not affected by ScyTx (Figure 3A). One can conclude that GFP–MgTx can be used as a component of the bioengineering KcsA–Kv1.3-based analytical system to search for and recognize Kv1.3 pore blockers of different origin. 

As demonstrated earlier, the bioengineering system based on a fluorescent ligand and spheroplasts expressing KcsA–Kv1.3 can be used to quantitatively estimate affinities of blockers that bind to the extracellular pore mouth [32]. Accordingly, GFP–MgTx was approbated in the competitive binding assay for the measurement of apparent dissociation constants (*K_ap_*) of the complexes of peptide blockers (ChTx, HgTx1) with the Kv1.3 binding site. The studied peptides compete with GFP–MgTx for the binding to KcsA–Kv1.3 in the subnanomolar concentration range (Figure 3C). The *K_ap_* constants of HgTx1 and ChTx, calculated using the Cheng–Prusoff Equation (3), were found to be 185 ± 6 and 240 ± 5 pM (mean *±* SEM, averaged over three independent experiments), respectively.

These *K_ap_* values for HgTx1 and ChTx correspond well with the values obtained for the same peptides using the KcsA–Kv1.3-based system with GFP-L1-HgTx1 as a fluorescent ligand: 200 and 115 pM, respectively [37]. Similar affinities were reported for HgTx1 and ChTx using other binding assays. Thus, the 50% blocking of rat Kv1.3 channels expressed in *Xenopus oocytes* was observed by voltage-clamp technique at 190 pM of ChTx [42]. The ^86^Rb^+^ flux induced by membrane depolarization was inhibited in eukaryotic cells by 50% at 38 pM of HgTx1 [43]. The results obtained demonstrate that GFP–MgTx as a component of the bioengineering KcsA–Kv1.3-based analytical system can be used to characterize affinities of the Kv1.3 pore blockers and select the most active ones for further physiological studies. 

## 3. Materials and Methods

### 3.1. Reagents and Cells

Ampicillin sodium salt, kanamycin sulfate, tetracycline hydrochloride, chloramphenicol, isopropyl β-D-1-thiogalactopyranoside, imidazole, sucrose, bovine serum albumin, Terrific Broth, EDTA, Na_2_HPO_4_, NaH_2_PO_4_, NaCl, KCl, MgCl_2_, and Tris(hydroxymethyl)aminomethane (Tris), were purchased from Sigma-Aldrich (Merck, Darmstadt, Germany). Restriction endonucleases KpnI and HindIII were from Thermo Fisher Scientific (Waltham, MA, USA). Plasmids pET23-MBP-L-MgTx [36] and pET23-GFP-L-AgTx2 [19] were derivatives of pET-23d vector (Novagen, Merck, Darmstadt, Germany). Plasmid pET28-KcsA–Kv1.3 was a derivative of pET-28a vector (Novagen), Merck, Darmstadt, Germany). E. coli strains Rosetta-gami B(DE3)pLysS and BL21(DE3) were obtained from Novagen (Merck, Darmstadt, Germany).

### 3.2. Gene Cloning, Expression and Purification of GFP–MgTx

A plasmid coding for GFP–MgTx was constructed by recombination of two previously obtained expression plasmids, pET23-MBP-L-MgTx [36] and pET23-GFP-L-AgTx2 [19]. For this, plasmid pET23-MBP-L-MgTx was cleaved by KpnI and HindIII restriction endonucleases, then DNA fragment encoding MgTx fused at its terminus with TEV cleavage site was purified by agarose electrophoresis and ligated with KpnI/HindIII-digested vector pET23-GFP-L-AgTx2. A scheme of cloning is shown in Figure 1A. The nucleotide sequence of a gene between KpnI and HindIII sites of the obtained plasmid pET23-GFP-L-MgTx was confirmed by sequencing using T7 terminator primer (Evrogen, Moscow, Russia).

Expression and purification of GFP–MgTx was carried out as described [36]. Briefly, *E. coli* Rosetta-gami B(DE3)pLysS cells were transformed with pET23-GFP-L-MgTx and cultivated at 37 °C in 50 mL of Terrific Broth in the presence of 100 mg/L ampicillin, 15 mg/L kanamycin, 12.5 mg/L tetracycline, and 34 mg/L chloramphenicol to the mid-log phase, then induced with 0.1 mM isopropyl β-D-1-thiogalactopyranoside and incubated at 20 °C for 20–22 h. After pelleting by centrifugation at 10,000× *g* for 20 min, the cells were disrupted by sonication in buffer A (46.6 mM Na_2_HPO_4_, 3.4 mM NaH_2_PO_4_ (pH 8.0), 300 mM NaCl, 10 mM imidazole), centrifuged at 36,000× *g* for 20 min, and the soluble fraction was loaded onto Ni Sepharose Fast Flow column (1.7 mL bed volume, GE Healthcare, Chicago, IL, USA). The column was successively washed with buffer A containing 10, 20, and 50 mM imidazole, the GFP–MgTx protein was eluted in the same buffer containing 150 mM imidazole, and imidazole was removed using PD-10 Desalting column (GE Healthcare, Chicago, IL, USA) in PBS (pH 7.4). The protein was stored at +4 °C in the dark in the presence of 0.01% sodium azide. Denaturing SDS-PAGE was used to analyze the protein purification steps (Figure 1C). Concentration of GFP–MgTx was determined by absorption spectroscopy using molar extinction coefficient for GFP (ε_488_ = 55,000 M^−1^cm^−1^). 

### 3.3. Recombinant Toxins

Recombinant peptides, namely, HgTx1, ChTx, and ScyTx, were obtained as described earlier [36].

### 3.4. Preparation of Spheroplasts and Binding Protocol

*E. coli* cells expressing KcsA or KcsA–Kv1.3 chimeric protein were cultured as described elsewhere [44]. Spheroplasts from *E. coli* cells were prepared according to the procedure described earlier [19]. In binding experiments, KcsA- or KcsA–Kv1.3-presenting spheroplasts were incubated (1000 cells μL^−1^, 2 h, 37 °C) with different concentrations of GFP–MgTx in a buffer containing 250 mM sucrose, 10 mM MgCl_2_, 10 mM Tris-HCl (pH 7.5), 4 mM KCl, 0.3 mM EDTA, and 0.1% bovine serum albumin. In competitive binding experiments, spheroplasts were incubated with GFP–MgTx (30 nM) and different concentrations of non-labelled peptide ligands (ChTx, HgTx1, ScyTx) or TEA (10 or 20 mM) for 2 h at 37 °C. 

### 3.5. Microscopy and Ligand–Channel Interaction Analysis 

Spheroplasts pre-incubated with non-labeled ligands and/or GFP–MgTx were transferred in a 12-well flexiPERM silicon chamber (Perbio, Aalst, Belgium) attached to a thin (0.16 mm) glass slide, and centrifuged at 200× *g* for 6 min to immobilize spheroplasts to the bottom of the wells for further microscopic analysis. The microscopic analysis was performed with a laser scanning confocal microscope LSM 710 (Zeiss, Oberkochen, Germany) using the α Plan-Apochromat 100×/1.46 oil immersion objective (Zeiss, Germany). The excitation and detection wavelengths of GFP–MgTx fluorescence were 488 nm and 495–590 nm, respectively. Binding of GFP–MgTx to spheroplasts was imaged with a laser scanning confocal microscopy and analyzed as described earlier [32]. Fluorescence intensity of GFP–MgTx bound to KcsA–Kv1.3 at the spheroplast membrane was estimated for each analyzed spheroplast using Image J software (National Institutes of Health, Bethesda, MD, USA) and averaged over spheroplast sampling (120–250 cells per point) to obtain the average fluorescence intensity *I_av_* of bound GFP–MgTx (mean ± SEM). 

Following the procedure described elsewhere [32], the dissociation constant (*K_d_*) of the complex between GFP–MgTx and KcsA–Kv1.3 was estimated from the measured *I_av_* dependence on the GFP–MgTx concentration using an equation:

*I_av_* ([*L*]) = *I_avs_* [*L*]/(*K_d_* + [*L*]) (1)

where [*L*] is a GFP–MgTx concentration, is *I_avs_* is the *I_av_* value at saturated binding. 

Competitive binding experiments were performed and analyzed as described earlier [32]. Briefly, in a typical competitive binding experiment, *I_av_* values were averaged over spheroplast sampling (150–300 cells per point) at a particular concentration of a competing ligand. A dependence of *I_av_* on the concentration of a competing ligand was analyzed to estimate the *IC_50_* concentration of a competing ligand that displaces 50% of GFP–MgTx from the complex with KcsA–Kv1.3. This dependence was analyzed using an equation:

*I_av_* = *I_m_*/(1+[*C*]/*IC_50_*) (2)

where [*C*] is the concentration of the added competing ligand, *I_m_* is *I_av_* at [*C*] = 0. 

Apparent dissociation constants (*K_ap_*) of non-labeled peptide blockers (ChTx, HgTx1) were calculated using the corresponding *IC_50_* values with the Cheng–Prusoff equation [32]:

*K_ap_* = *IC_50_*/(1 + [*L_f_*]]/[*K_d_*]) (3)

where *K_d_* is the dissociation constant of the complex between GFP–MgTx and KcsA–Kv1.3. [*L_f_*] is the concentration of free GFP–MgTx, which is assumed to be equal to a concentration of added GFP–MgTx (30 nM), because [*L_f_*] is considerably higher than the concentration of bound GFP–MgTx at the conditions of our experiments [32]. 

It should be clarified that affinity of a non-labeled blocker is characterized with *K_ap_* instead of the dissociation constant in order to highlight that it is determined by an indirect method in the competitive binding experiment. 

The *K_d_* value of GFP–MgTx and *K_ap_* values of other studied blockers were averaged over three independent experiments and presented as mean ± SEM, *n* = 3.

Significance of differences between *I_av_* data was estimated with the unpaired t-test calculating two-tailed *p* value. The differences were considered to be significant if *p* < 0.05.

### 3.6. Optical Spectroscopy 

Absorption and fluorescence spectra of GFP–MgTx were measured in phosphate-buffered saline using Cary 50 spectrophotometer and Cary spectrofluorimeter (Varian, CA, USA), respectively.

## 4. Conclusions

Peptide blockers of Kv1-channels fused with fluorescent proteins are an attractive alternative to radioligands and fluorescent ligands labeled with organic dyes. They can be produced easily and inexpensively using protein bioengineering approaches. 

The developed GFP–MgTx is demonstrated to be a fluorescent ligand having nanomolar affinity to the external binding site of pore blockers of the Kv1.3 channel. GFP–MgTx can be used as a component of an analytical system based on the hybrid KcsA–Kv1.3 channel for screening, assessment of affinity and pre-selection of Kv1.3 blockers for their subsequent study as potential drugs for the treatment of autoimmune and neurological disorders. 

Data obtained for GFP–MgTx together with those reported for GFP-AgTx2, GFP-OSK1, and GFP-HgTx1 [18,19,37] show that the proposed design of chimeric GFP-tagged proteins is well suited for creating a variety of FP-Tx constructs aiming to discover the advanced fluorescent probes of ion channels. 

## Data Availability

The data presented in this study are available on request from the corresponding author. The data are not publicly available due to local regulations.

## Abbreviations

AgTx2	agitoxin 2
GFP	green fluorescent protein
MgTx	margatoxin
HgTx1	hongotoxin 1
ChTx	charybdotoxin
ScyTx	scyllatoxin
TEA	tetraethylammonium
*IC_50_*	a concentration of a competing ligand that displaces 50% of the fluorescent probe from the complex with the receptor
*K_d_*	dissociation constant of GFP–MgTx
*K_ap_*	apparent dissociation constant of non-labeled ligand determined in the competitive binding experiment
*I_av_*	average fluorescence intensity of GFP–MgTx bound at a surface of a spheroplast expressing KcsA–Kv1.3
*I_avs_*	*I_av_* value at saturated binding of GFP–MgTx
*I_m_*	*I_av_* value at zero concentration of a competing ligand in the competitive binding experiment
